# Review of Medical Studies on COVID-19 During the Pandemic Period

**DOI:** 10.5152/eurasianjmed.2022.22336

**Published:** 2022-12-01

**Authors:** Alperen Aksakal, Buğra Kerget

**Affiliations:** 1 Department of Pulmonary Diseases, Atatürk University Faculty of Medicine, Erzurum, Turkey

**Keywords:** COVID-19, review, pandemic

## Abstract

Due to the COVID-19 pandemic, both the university hospital and the city hospital have faced a significant patient load in our city. During this period, academic articles were written that contributed significantly to the literature on both hospitals struggling with patient density. In our study, we aimed to compile medical articles about COVID-19 in our city using the Web of Science and PubMed database.

**Main Points**

- During the COVID-19 process, important studies that can contribute to the literature have been carried out in our region, and a large part of these studies are clinical studies.
- These studies have been cited 69 times in the last 3 years and SCI-E journals constitute a significant portion of the citations.
- During the pandemic period, all clinics followed COVID-19 patients in both intensive care units and wards due to the increasing number of cases. This has led to the emergence of important studies in many clinical branches, particularly in pulmonology and infectious diseases.

The pneumonia outbreak that began in December 2019 in Wuhan, the capital of China's Hubei province, was named coronavirus disease 2019 (COVID-19) by the World Health Organization (WHO). Following an increase in the number of positive infected cases in China, on January 30, 2020, the WHO declared this viral outbreak a pandemic.^1^ COVID-19 is a disease characterized by respiratory distress, fever, cough, fatigue, pneumonia, and muscle pain; it can be asymptomatic or affect vital organs. Especially in some patient groups, endothelial damage may develop in many vital organs, especially the lungs, due to the overproduction of proinflammatory cytokines. Proinflammatory cytokines synthesized by T lymphocytes and macrophages play an important role in this so-called cytokine storm.^2^

In our review, we searched PubMed and Web of Science for medical studies related to COVID-19 conducted in Erzurum province and limited the search to the last 3 years. In our study, summaries of 29 studies were presented. The Web of Science database was also used to search the citations of articles and case reports within the last 3 years and self-citations were excluded.

In studies by Kerget et al.^3-5^ serum alpha defensin, interleukin (IL)-1Ra, IL-18, surfactant protein D, IL-6, and kidney injury molecule 1 (KIM-1) levels were found to be significantly higher in patients with macrophage activation syndrome (MAS) and acute respiratory distress syndrome (ARDS) compared to patients without. In the web of science search for these 3 studies, it was observed that the publications received 34 citations in total. Again, a significant portion of the studies were COVID-19 publications and it was observed that the study results were blinded to the results found.^6-39^ In a similar study, a positive correlation was observed between serum migration inhibitory factor level and disease severity.^40^ A total of 7 citations were observed for this study. Unlike other studies, these citations were observed in COVID-19 patients as well as in cardiovascular diseases, individuals with trypanosome infection, and other viral infections.^41-47^ Furthermore, in another study, the trigger receptor (TREM)-1/TREM-2 ratio expressed in myeloid cells was found to be significantly higher in the severe patient group.^48^ There are 3 citations in total for this study. While 2 of the citations were for COVID-19 infection, 1 study was to examine the role of TREM-2 in infection.^49-51^ In another study, it was revealed that low endogenous carboxyhemoglobin (COHb) level in blood gas at the time of hospital admission was associated with ARDS, MAS, and mortality in COVID-19.^52^ It was determined that this study, which investigated the place of CoHb level in predicting the course of COVID-19, was cited 4 times. Again, all of these citations were COVID-19 publications. In a study, it was found that CoHb levels were higher in patients with bacterial sepsis than in patients hospitalized due to COVID-19.^53-56^ In a study investigating the relationship between insulin-like growth factor-binding protein 5 (IGFBP5) expression and plasma osteopontin (OPN) levels and disease severity in COVID-19 patients, high OPN levels and low IGFBP5 levels were found to be associated with disease severity.^57^ In a study examining the relationship between monocyte chemoattractant protein-1 and surfactant protein A levels and the clinical course and prognosis of COVID-19, both biomarkers showed a positive correlation with disease severity.^58^ In a similar study, serum chitinase 3-like protein-1 (CHI3L1) and IL-6 levels were positively correlated with the clinical course of COVID-19 infection.^59^ In a study examining the relationship between adropin levels and poor prognosis in diabetic patients diagnosed with COVID-19, adropin levels were negatively correlated with C-reactive protein (CRP), ferritin, and d-dimer levels.^60^ It was also observed that there were 2 citations in the literature for this study. One of the publications was on adropin levels in patients with acute pulmonary thromboembolism, while the other study was planned in patients with metabolic syndrome.^61,62^ In a study examining the usefulness of serum transforming growth factor-beta 1 (TGF-beta 1) and connective tissue growth factor (CTGF) levels in predicting disease severity in COVID-19 patients with lung involvement, it was concluded that TGF-beta 1 and CTGF are potential biomarkers that can distinguish COVID-19 patients with lung involvement and indicate disease severity.^63^

In the study examining serum trace element levels in COVID-19 patients, it was found that serum Zn and Se concentrations were significantly lower in COVID-19 patients compared to healthy individuals.^64^ It was observed that there were 5 citations for the study and all of the studies were also conducted on COVID-19 patients.^65-69^ Another study concluded that vitamin D supplementation has the potential to reduce the incidence, severity, and risk of death from pneumonia caused by the cytokine storm of many viral infections, including COVID-19.^70^ In a study investigating the relationship of progranulin level with the severity of the disease in COVID-19, it was concluded that progranulin reached high levels in COVID-19 and may be a better biomarker than CRP in diagnosis and prognosis.^71^ In a study examining galectin-3 levels in COVID-19 patients, it was revealed that galectin-3 may be a diagnostic tool for typical pneumonia associated with COVID-19 and an indicator of the severity of pneumonia.^72^ In a study investigating the effects of laboratory parameters on the prognosis of COVID-19, it was concluded that an increase in CRP, procalcitonin, erythrocyte sedimentation rate, ferritin, troponin, d-dimer, lactate dehydrogenase, and neutrophil count and a decrease in albumin, platelet, and lymphocyte count were associated with the severity of the disease.^73^ In a similar study, prognostic laboratory parameters for COVID-19 were investigated in a group of geriatric patients with high mortality risk, and it was concluded that a high neutrophil/lymphocyte ratio was an independent risk factor for mortality.^74^ While 1 citation was observed for this study, it was on risk factors in patients hospitalized in the intensive care unit.^75^

Genetic studies to predict the severity of the disease in COVID-19 are also available and a study investigated the association between pentraxin 3 (PTX3) gene polymorphisms rs2305619 (281A/G) and rs1840680 (1449A/G) and the development of MAS. Analysis of the PTX3 1449A/G polymorphism in COVID-19 patients showed that individuals with the AG genotype were relatively more protected from MAS compared to individuals with the AA genotype. Lower serum PTX3 levels were also observed in patients carrying the A allele.^76^ While there were 4 citations for the study, the majority of the citations were COVID-19 studies and only 1 study was on PTX-3 metabolism.^77-80^ Another genetic study aimed to evaluate the relationship between IL-6 gene polymorphisms—174G/C and—597G/A and the course of COVID-19, and analysis of the -174G/C polymorphism in patients with MAS revealed that the G allele may be a risk factor for increased serum IL-6 levels and progression to MAS.^81^ This study on the genetic predisposition of COVID-19 was cited 6 times and a significant portion of the publications was also on COVID-19. Only 1 study was aimed at determining the frequency of genetic polymorphisms in the Turkish population.^82-87^ In addition, genetic studies have shown that PAPP-A, STC-2, and HIF-1α gene expression is higher in patients with a severe clinical course.^88^ In a similar study examining CYP2E1 and caspase-3 gene expression in patients with COVID-19 infection, it was found that caspase-3 expression increased but CYP2E1 expression decreased in COVID-19 patients compared to healthy individuals.^89^

In a study investigating the relationship between exhaled CO levels and parenchymal involvement in patients hospitalized due to COVID-19 pneumonia, the exhaled CO levels of the patients in the study were examined at the time of hospitalization and it was found that exhaled CO levels were higher in patients who developed MAS.^90^ A similar study aimed to evaluate the relationship between the severity and parenchymal involvement of COVID-19 and exhaled nitric oxide (FeNO) levels. As a result of the study, a positive correlation was observed between exhaled FeNO level and disease severity and computed tomography (CT) uptake.^91^ There is only 1 reference to this study on exhalation tests and the study was on the relationship between alveolar nitric oxide concentration and COVID-19 severity.^92^ In a study evaluating the relationship between laboratory parameters and pulmonary function tests in COVID-19 patients, it was shown that the decrease in CRP from admission to day 7 of treatment was correlated with an increase in forced expiratory volume estimated at 1 second (FEV_1_) and forced vital capacity (FVC) in the same period. In the same study, a decrease in fibrinogen levels was also correlated with an increase in FEV_1_ and FVC.^93^ In the study evaluating the effectiveness of high-frequency chest wall oscillation in patients with COVID-19 pneumonia, it was shown that pulmonary rehabilitation applied in the early period with a high-frequency chest wall oscillation device contributed to the improvement of pulmonary function tests and oxygenation.^94^ In the study examining the effect of montelukast treatment on clinical course, pulmonary function test, and mortality in COVID-19 patients, it was observed that lactate dehydrogenase, fibrinogen, d-dimer, CRP, and procalcitonin levels were significantly lower at the end of day 5 in the patient groups who used montelukast for 5 days compared to the other groups. In addition, a significant increase in FEV_1_ level was observed in the pulmonary function test performed on day 5 in this study.^95^

In a study evaluating the 3-month post-treatment follow-up of COVID-19 patients, patients with severe pneumonia requiring intensive care hospitalization had lower percent FVC (%FVC), percent FEV1 (%FEV1), and the ratio of predicted diffusion capacity of the lungs for carbon monoxide to alveolar volume (%DLCO/VA) than patients with the milder clinical course at the 3-month follow-up. In addition, this study showed significant improvement in radiologic findings in all patient groups at week 12.^96^ There were 2 references to this study on COVID-19 long-term patient follow-up, one of the studies was on long-term COVID-19 patients, while the other was on risk factors in COVID-19.^97,98^ In a similar study, COVID-19 evaluated the relationship between CRP/albumin ratio and pulmonary function tests at 12 weeks after treatment. In this study, it was revealed that CRP/albumin ratio was negatively correlated with oxygen saturation (SO2), %FEV1, %FVC, and %DLCO.^41^ In a study investigating the efficacy of anti-fibrotic treatment in the post-COVID-19 period, a significant increase was observed in FVC, FVC%, FEV1, FEV1%, DLCO%, DLCO/VA%, 6-minute walk test, and room air saturation levels at week 12 in all patients given antifibrotic treatment.^99^

## Conclusion

As a result, important studies that can contribute to the literature have been carried out in our region during the COVID-19 process and all of these studies were clinical studies. These studies have been cited 69 times in the last 3 years, and SCI-E journals constitute a significant portion of the citations. During the pandemic period, all clinics followed COVID-19 patients both in intensive care units and wards due to the increasing number of cases. This has led to the emergence of important studies in many clinical branches, particularly in pulmonology and infectious diseases.
